# Vaccination and Government Stringent Control as Effective Strategies in Preventing SARS-CoV-2 Infections: A Global Perspective

**DOI:** 10.3389/fpubh.2022.903511

**Published:** 2022-06-24

**Authors:** Peng Yang, Zhe Yang, Chenxi Zhao, Xinrui Li, Zhongjun Shao, Kun Liu, Lei Shang

**Affiliations:** ^1^Department of Health Statistics, Ministry of Education Key Lab of Hazard Assessment and Control in Special Operational Environment, School of Public Health, Air Force Medical University, Xi'an, China; ^2^Department of Epidemiology, Ministry of Education Key Lab of Hazard Assessment and Control in Special Operational Environment, School of Public Health, Air Force Medical University, Xi'an, China; ^3^School of Public Health, Baotou Medical College, Baotou, China; ^4^School of Medicine, Northwest University, Xi'an, China

**Keywords:** COVID-19, SARS-CoV-2, vaccination, stringency index, interrupted time series model

## Abstract

With the rapid implementation of global vaccination against the coronavirus disease 2019 (COVID-19), the threat posed by the disease has been mitigated, yet it remains a major global public health concern. Few studies have estimated the effects of vaccination and government stringent control measures on the disease transmission from a global perspective. To address this, we collected 216 countries' data on COVID-19 daily reported cases, daily vaccinations, daily government stringency indexes (GSIs), and the human development index (HDI) from the dataset of the World Health Organization (WHO) and the Our World in Data COVID-19 (OWID). We utilized the interrupted time series (ITS) model to examine how the incidence was affected by the vaccination and GSI at continental and country levels from 22 January 2020 to 13 February 2022. We found that the effectiveness of vaccination was better in Europe, North America, and Africa than in Asia, South America, and Oceania. The long-term effects outperformed the short-term effects in most cases. Countries with a high HDI usually had a high vaccination coverage, resulting in better vaccination effects. Nonetheless, some countries with high vaccination coverage did not receive a relatively low incidence due to the weaker GSI. The results suggest that in addition to increasing population vaccination coverage, it is crucial to maintain a certain level of government stringent measures to prevent and control the disease. The strategy is particularly appropriate for countries with low vaccination coverage at present.

## Introduction

It has been more than 2 years since the outbreak of the coronavirus disease 2019 (COVID-19). Although the increased vaccine coverage has relieved the threat posed by the pandemic, it remains a most serious public health problem in the world. As of 13 February 2022, 412 million COVID-19 cases have been reported worldwide, resulting in a total of 5.8 million deaths, whereas, at the end of 2020, it was only 83 million infections and 1.8 million deaths (1).

As an emerging respiratory infectious disease, the pathogen of severe acute respiratory syndrome coronavirus 2 (SARS-CoV-2) is characterized by rapid mutation and rapid transmission (2). To date, 12 different mutant strains from Alpha to Omicron have been discovered and reported (3). Since the end of last year, the Omicron variant has caused a significant increase in infectivity. Fortunately, vaccination drivers have been proven to be an effective method for the disease (4–7). The incidence of COVID-19 fell gradually after countries (Canada, China, Israel, Latvia, Norway, United Kingdom, the United States, and Russia) started vaccinations and were followed by later vaccinations in other countries. As of 13 February 2022, the global full vaccination coverage rate (VCR) has reached up to 54.2%, while at the end of 2020, it was only 0.0005%. Meanwhile, we found that not all countries with high vaccination coverage showed ideal effects. Despite better economic levels usually having higher vaccination coverage, many countries still maintain high incidence, even higher than in those countries with low vaccination coverage. As a result, the prevention of COVID-19 cases is not only related to population vaccination but also depended on government control measures (e.g., public event cancellations, gathering restrictions, public transportation shut down, and quarantine requirements). As shown by the reported experience of addressing SARS-CoV-2, countries with stricter prevention and control measures are better at containing the epidemic (8–10).

Studies have suggested that when the VCR reached a certain threshold, herd immunity would be sufficient for protecting the population against infections of SARS-CoV-2. Specifically, when the reproduction rate (R_0_) is set to 2.5 for COVID-19 (11, 12), the coverage threshold for herd immunity needs to be 60.0%. Since vaccine efficacy (VE) varies from different manufacturers, it must be at least 69% to form the infection barrier. However, 72.1% of countries worldwide do not currently meet the minimum threshold for reaching herd immunity. Notably, about 48.3% of countries have a VCR of <50%, and most of these countries are the developing countries. Therefore, it is imperative to clarify the relationships between the vaccination, government control measures, human development index (HDI), and the incidence of COVID-19 from a global perspective. Previous studies have analyzed the relationship between COVID-19 incidence, vaccination, and government control measures (8–10, 13–18). However, these studies mostly focused on local areas or individual countries, and there are no worldwide reports based on long-term data to examine VE and stringent measures concurrently.

We collected COVID-19 daily cases, vaccinations with the HDI, and government stringency index (GSI) of 216 countries from 22 January 2020 to 13 February 2022. The study conducted a comprehensive analysis using an interrupted time series (ITS) model to examine how the incidence was affected by the vaccination and GSI at continental and country levels.

## Materials and Methods

### Data

We collected 216 countries' COVID-19 data from 22 January 2020 to 13 February 2022. The main daily indices included new cases, new vaccinations (new vaccination doses administered), people vaccinated (total number of people who received at least one vaccine dose), people fully vaccinated (total number of people who received all doses prescribed by the vaccination protocol), and the GSI. The indices for countries were total population and HDI. To avoid daily fluctuation, we converted data from days to weeks, for a total of 108 weeks. We excluded countries with fewer than 1,000 total cases to fit the model, leaving 178 countries in the final analysis.

The daily data of COVID-19 new cases, new vaccinations, and related indices were obtained from the dataset of the World Health Organization (WHO) (1) and Our World in Data COVID-19 (OWID) (19). GSI data were collected from the Blavatnik School of Government, University of Oxford (20, 21), and HDI data were acquired from the United Nations Human Development Report (22).

Government stringency index is data of time series. Each day has a specific number to reflect the extent of control measures. The values vary from 0 to 100, with 100 indicating the strictest response. It is a composite measure based on 20 government response indices: school closures, workplace closures, public event cancellations, gathering restrictions, public transportation shut down, stay at home requirements, restrictions on internal movement, international travel controls, income support, debt/contract relief, fiscal measures, international support, public information campaigns, testing policy, contact tracing, emergency investment in healthcare, investment in vaccines, facial coverings, vaccination policy, and protection of elderly people.

The HDI is a comprehensive summary index that measures economic and social development levels in three essential dimensions of human development: a long and healthy life, being knowledgeable, and a decent standard of living. The detailed indices include life expectancy at birth, expected years of schooling, mean years of schooling, and gross national income per capita. HDI scale runs from 0 to 1, with 1 representing the best development possible. A higher HDI indicates the better socioeconomic, welfare, and security situations in the country. Each country has a single HDI value.

### Baseline ITS Model

We used the ITS model (23–28) to estimate the impact of vaccination on COVID-19 incidence. Three variables (*time*_*t*_, *vax*_*t*_, and *post vax*_*t*_) are in the baseline ITS model as follows:


(1) $$\begin{array}{l} Y_{t}=log(population)+\beta_{0}+\beta_{1}\times time_{t}+\beta_{2}\times vax_{t}\\ \;+\beta_{3}\times post\;vax_{t}+\varepsilon_{t} \end{array}$$

where *Y*_*t*_ denotes the weekly number of cases in continents/ countries, β_0_ represents the baseline level at the beginning of the time series, and β_1_ represents the pre-vaccination trend where *time*_*t*_ is set as 1 (first week) to 108 (last week of the full-time series) or less (countries with late-onset of COVID-19 cases). Where β_2_ estimates the change in level between pre- and post-vaccination through the dummy variable *vax*_*t*_, wherein *vax*_*t*_ = 0 signifies pre-vaccination and *vax*_*t*_ = 1 post-vaccination. β_3_ estimates the slope change after vaccination, wherein *post vax*_*t*_ is coded as 0 before vaccination and is accumulated from 1 since the vaccination. ε_t_ is the error term, which is the remaining part after removing the effect of the variables β_1_, β_2_, and β_3_ on *Y*_*t*_ in the equation. The smaller the value, the better the fit of the equation. In the study, the *quasi-Poisson* was used in the ITS to avoid overdispersion of long time series data by conventional linear methods. *log (population)* was included in the model as an offset variable to ensure the comparability of various countries with different population sizes. Moreover, the model was adjusted for seasonality by using Fourier transform (*Harmonic* term in R) to specify the number of sine and cosine pairs, and the length of a period (i.e., 4 weeks as a month).

### Improved ITS

We added the *GSI* as an adjusted variable based on the model presented in Equation (1) to equalize the impact of government stringent control measures on the incidence of pre- and post-vaccinations among countries in Equation (2). Due to a delay between GSI and disease onset, we set lag = 1 week (7 days) for *GSI* (29) in the model. In addition to that, we used the variable *VCR*_t_ instead of *post vax*_*t*_ to examine the effect on the incidence with the increase of the vaccination coverage in Equation (3).


(2) $$\begin{array}{l} Y_{t}=log(population)+\beta_{0}+\beta_{1}\times time_{t}+\beta_{s}\times vax_{t}\\ \;+\beta_{3}\times post\;vax_{t}+\beta_{4}\times GSI_{t-1}+\varepsilon_{t} \end{array}$$

(3) $$\begin{array}{l} Y_{t}=log(population)+\beta_{0}+\beta_{1}\times time_{t}+\beta_{\text{2}}\times vax_{t}\\ \;+\beta_{l}\times VCR_{\text{t}}\text{+}\beta_{4}\times GSI_{\text{t-1}}+\varepsilon_{t} \end{array}$$

In Equations (2) and (3), β_4_ estimates the effect of the GSI on the disease incidence. β_s_ denotes the short-term (just vaccinated) impact of vaccination because *vax*_*t*_ as a dummy variable indicates the change of pre-vaccination and onset of vaccination. β_*l*_ denotes the long-term impact of vaccination since *VCR*_*t*_ is accumulated over weeks. Other variables are defined in the same way as in Equation (1).

### Event Study

The advantage of the ITS is to design the treatment (post-vaccination) and control (pre-vaccination) groups and compare the difference between the two groups by constructing a dummy variable. Even if the results show a decline in disease incidence after the implementation of vaccination, the result may not be driven by the vaccination but by systematic differences. Nevertheless, we can still examine the trends by moving the start time of implementation ahead and backward by a few weeks, respectively, and observe whether treatment and control groups are comparable among these weeks. To do this, we conducted an event study by adding the parameter *k* into the model based on Equation (1) and fitted the following equation:


(4) $$\begin{array}{l} Y_{\left(t,k\right)}=log(population)+\beta_{0}+\beta_{1}\times time_{t,k}+\beta_{2}\times vax_{t,k}\\ \;+\beta_{3}\times post\;vax_{t,k}+\varepsilon_{t} \end{array}$$

In the study, we set *k* ∈ [−5, 9] (*k* = 0 indicates the week of vaccination implementation) to examine the overall trend of 15 weeks. When *k* ∈ [−5, 0), the model compares the trends of VEs among 5 weeks (just over a month) before the vaccination. When *k* ∈ (0, 9], the model compares the trends among 9 weeks (just over 2 months) after vaccination.

In the data analysis process, we first estimated and compared VEs on the COVID-19 incidence among continents and countries using Equations (2) and (3). We also observed the changing trend of the VEs between pre- and post-vaccinations using Equation (4). We further investigated the relationships between estimated VEs, vaccination rate, and HDI across countries by Spearman correlation (variables with abnormal distribution) and Pearson correlation (variables with normal distribution). Lastly, we observed the corresponding position of each country's average stringency index, cumulative incidence, and VCR through the two and three-dimensional graphs. Meanwhile, we compared the differences of VEs among continents and VEs classified by HDI quantiles (25, 50, and 75%) by utilizing the Wilcoxon and Kruskal-Wallis H test. All the analyses were performed in R software (version 4.1.2). *p* < 0.05 indicates a statistical difference.

## Results

### Global Distribution and Status of COVID-19 Incidence, VCR, and GSI

**Figure 1:**
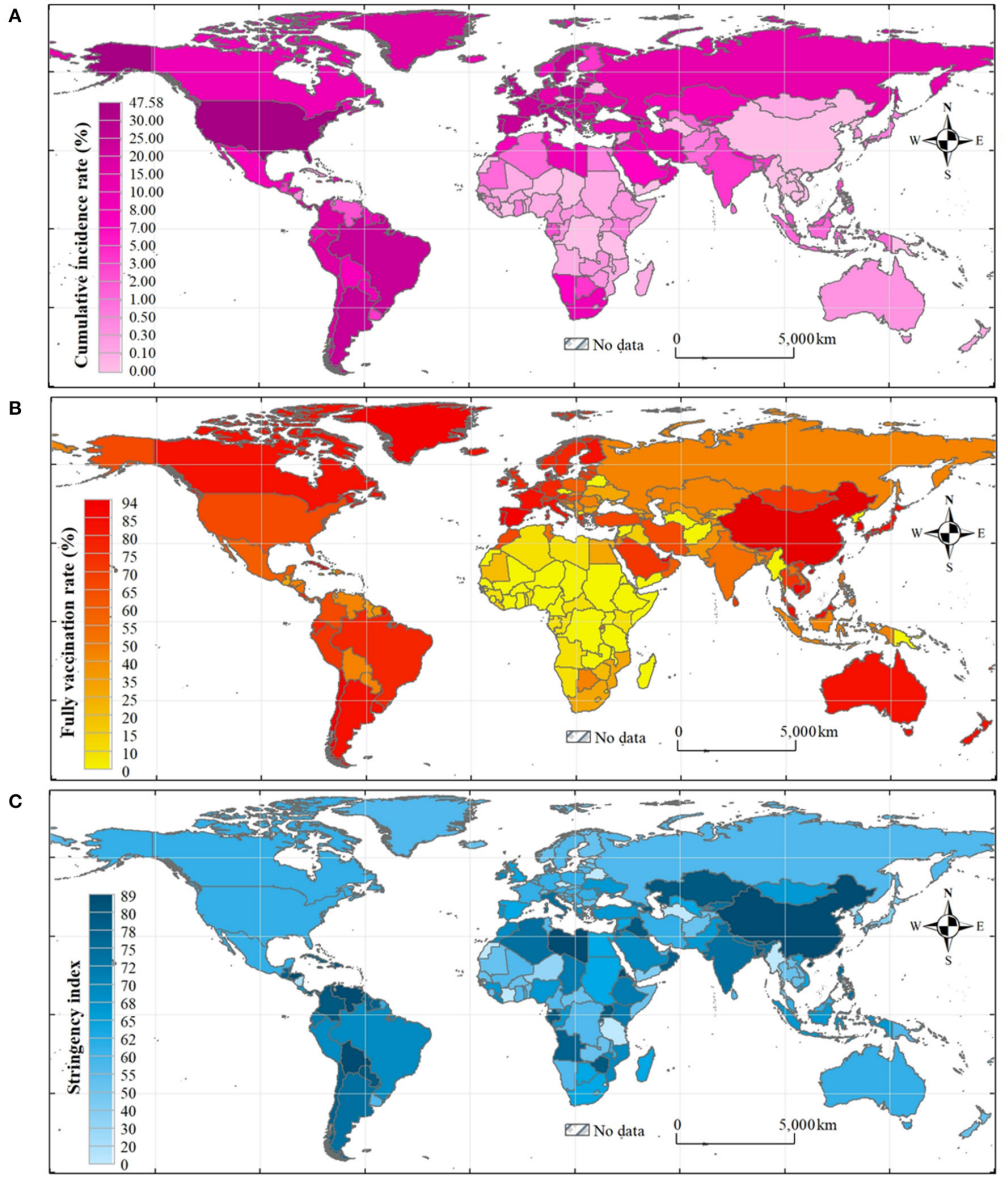
Global distributions of cumulative incidence rate **(A)**, fully vaccination rate **(B)**, and stringency index **(C)**.

As of 13 February 2022, the top 10 countries worldwide with the highest cumulative incidence rate (mean value: 10.1%, range: 0.007–49.7%) of COVID-19 in sequence are the Faeroe Islands, Andorra, San Marino, Seychelles, Slovenia, Denmark, Israel, Georgia, France, and Slovakia; the top 10 countries with the highest VCR (52.2%, 0.05–98.9%) are the United Arab Emirates, Portugal, Cuba, Brunei, Chile, Singapore, Malta, China, Spain, and Argentina; and the 10 countries with highest average GSI (54.9, 10.7–79.2) are Honduras, Venezuela, Myanmar, Eritrea, Jamaica, Iraq, Suriname, Bangladesh, Palestine, and Libya. The global distributions of the three indices are shown in Figure 1.

**Figure 2:**
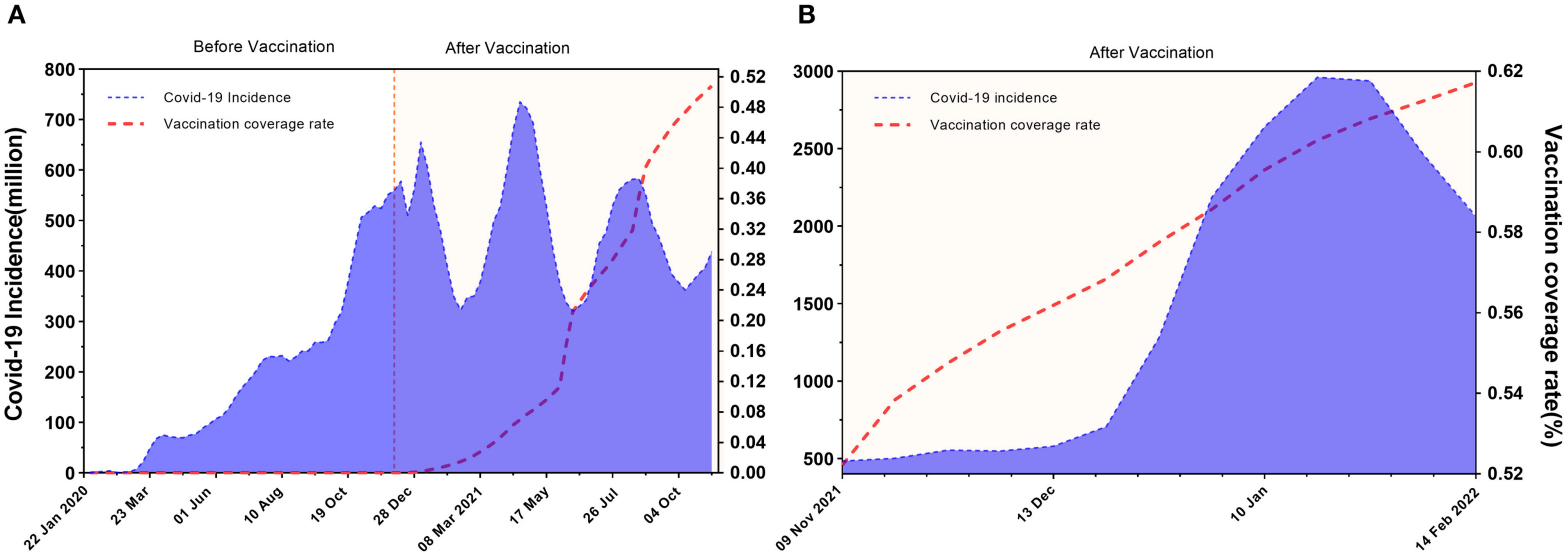
Global trends of COVID-19 incidence gradually decline as vaccination coverage increases. The period for **(A)** is from 22 January 2020 (first SARS-CoV-2 case worldwide) to 8 November 2021 (before Omicron onset). **(A)** shows that the incidence gradually decreases after countries began vaccination at week 47 (7–13 December 2020). The third peak of incidence (week 84) is much lower than the previous two peaks. The range for **(B)** is from 9 November to 13 February 2022 (Omicron onset till today). **(B)** reveals even if the Omicron incidence was considerably larger than other mutant strains from 9 November to 13 February 2022, the global incidence began to decline on 30 January 2022 after a short peak.

Figure 2 illustrates the global trend of COVID-19 incidence fluctuation along with the period from 22 January 2020 to 13 February 2022. Since the incidence of the Omicron virus was considerably higher than that of the original and other mutant strains, Figure 2 is divided into two figures, i.e., Figure 2A shows the time period from 22 January 2020 (first SARS-CoV-2 case worldwide) to 8 November 2021 (before Omicron onset). After countries started vaccinations at week 47 (7–13 December 2020), COVID-19 incidence gradually decreased. The third peak of incidence (week 84) is much lower than the previous two peaks. Figure 2B depicts the time span from 9 November 2021 to 13 February 2022 (Omicron onset till today). As the VCR continued to climb, the incidence began to fall on 30 January 2022 after a short peak.

### Impact of Vaccination on COVID-19 Incidence Among Continents

**Figure 3:**
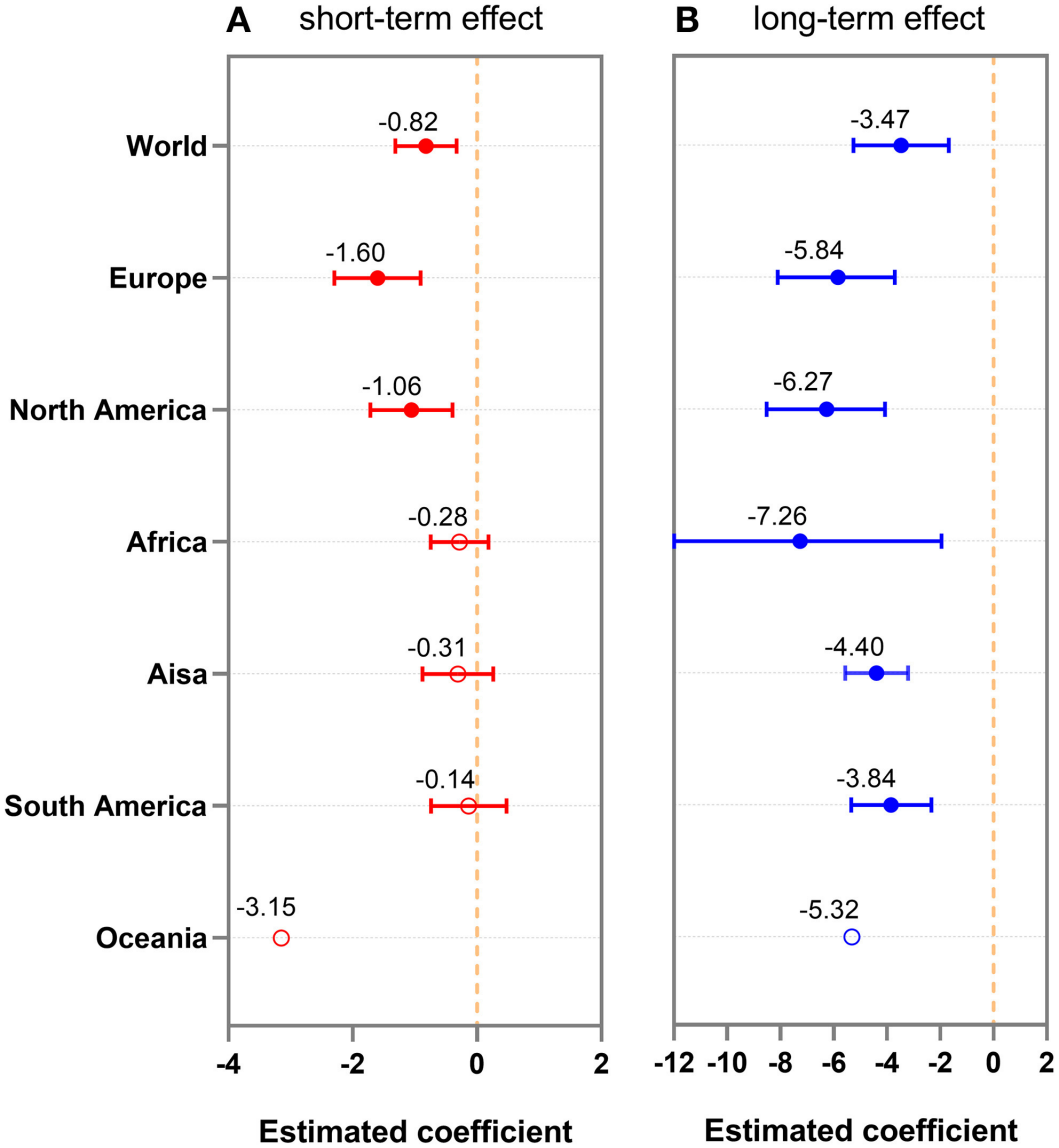
Shot-term **(A)** and long-term **(B)** impacts of vaccination on COVID-19 cases among continents. Red circles are filled if the value is significantly different from the null distribution (*p* < 0.05), and open otherwise. The number denotes the median, and error bars denote quantiles 0.025 and 0.975. Figures show that vaccine efficacies (VEs) in Europe, North America, and Africa were higher than that in Asia and South America. VEs between countries varied more in Africa (a wider error bar) but were balanced in other continents (a narrower error bar).

The impact of vaccination on the COVID-19 incidence among continents was estimated through Equations (2) and (3). Figure 3 shows that the long-term VEs are generally better (smaller estimated coefficients) than the short-term VEs (*p* < 0.05) in all six continents and worldwide. VEs were significantly higher in Europe and North America than that in other continents for the short-term effect (Figure 3A; *p* < 0.05). Long-term VEs in Europe, North America, and Africa were generally higher than in Asia, South America, and Oceania (Figure 3B; *p* < 0.05). Furthermore, VEs varied more across countries in Africa (a wider error bar) and were more balanced in other continents (a narrower error bar).

We divided variants into two types according to the characteristics and onset time of SARS-CoV-2 variants. One type is Alpha, Beta, Delta, and other variants (lower transmission efficiency, higher rate of severe illness) and the other is the Omicron variant (higher transmission efficiency, lower rate of severe illness). We further analyzed the short-term and long-term impacts of vaccination on COVID-19 cases among continents between two types of variants, respectively (Supplementary Figure 1). We found that group analysis results were mostly consistent with the results of the overall analysis. Long-term VEs were generally better than short-term VEs in all six continents and worldwide. VEs were significantly higher in Europe, North America, and Africa than in other continents.

**Figure 4:**
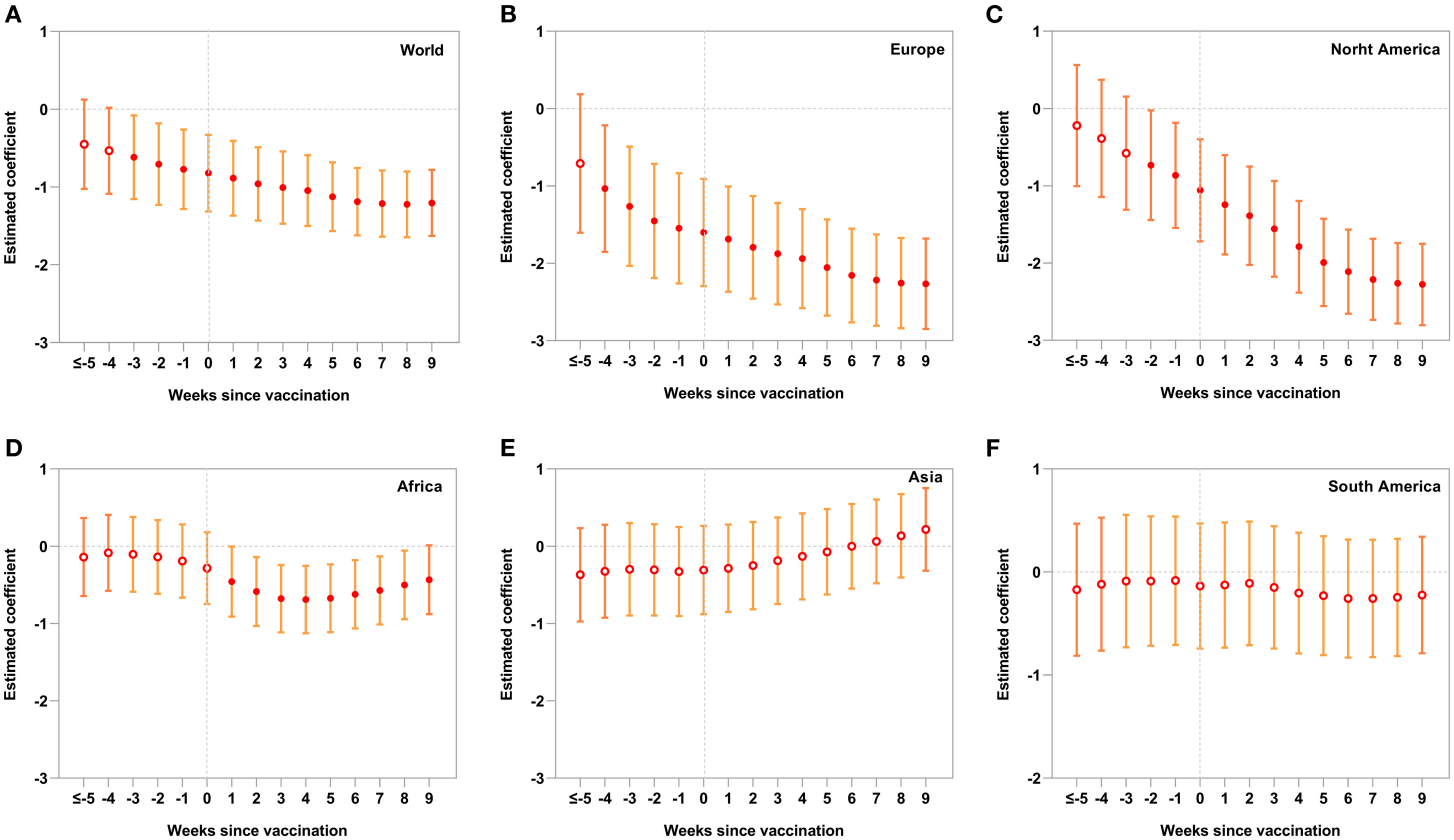
Event-study results on short-term effects of vaccination on COVID-19 cases. We fit the model by ranging *k* from −5 to 9 as the start of the intervention in Equation (4). Each column **(A–F)** represents a separate interrupted time series (ITS) model. The estimated coefficients are plotted along with their 95% confidence intervals (CIs). Vertical dotted lines indicate the week since vaccinations. Red circles are filled if the values are significant (*p* < 0.05), and open otherwise. Figures show that VEs gradually improved (a smaller estimated coefficient) and stabilized (a narrower confidence interval) in the World, Europe, and North America over time. In Africa, VEs were gradually increased and changed from non-significance to significance since the vaccination.

We set *k* from −5 to 9 individually as the start of the intervention in Equation (4) and fitted the models to investigate their trends over 15 weeks (about 3 months). Figure 4 shows that since the vaccination rollout (*k* = 0), VEs in the World, Europe, and North America gradually increased over time, peaking at around 8 weeks (2 months). VEs were also more and more stable (confidence intervals became narrower and narrower) over time. VEs rose and moved from non-significance to significance in Africa since the vaccination.

### Impact of Vaccination on COVID-19 Incidence Among Countries

We compared the impact of vaccination on the incidence across countries by Equations (2) and (3) and found that not all countries showed effectiveness as expected after vaccination implementation. An effective vaccination (estimated coefficient <0 and *p* < 0.05) was only found in 84.9% of the short-term and 85.1% of the long-term for all countries. Specifically, Europe and North America had a significantly higher proportion (>90%) of countries with short-term effects, followed by Africa and Asia (*p* < 0.05). Europe still had a higher proportion (>90%) of countries with long-term effects, followed by Africa (*p* < 0.05; Supplementary Figure 2).

**Figure 5:**
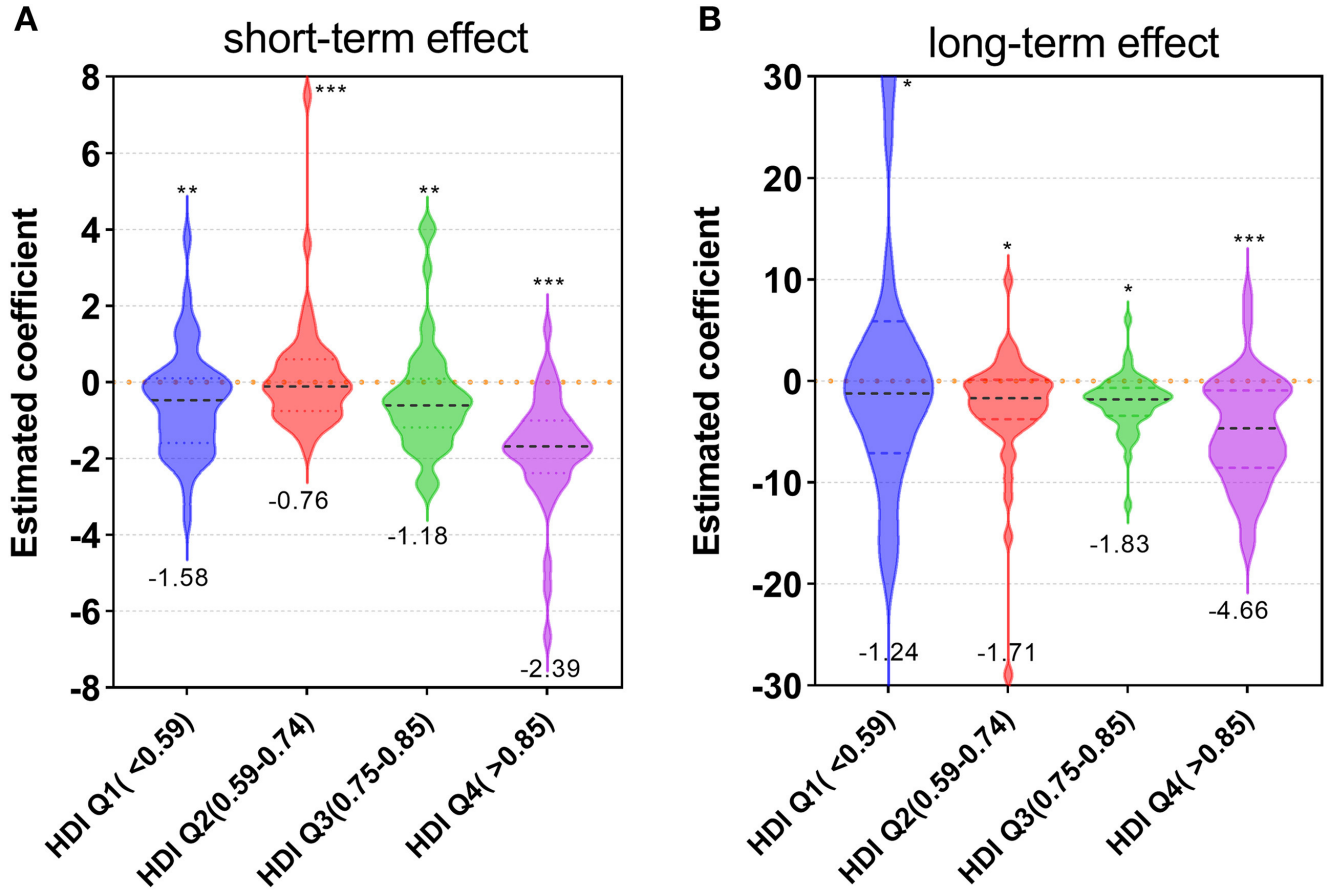
Estimated coefficients of short-term **(A)** and long-term **(B)** effects divided by human development index (HDI) quantiles. *** means the group has a significant difference from the other three groups, ** means from the other two groups, and * means from the other group. Solid lines and numbers denote media value, and dotted lines denote quantiles 0.25 and 0.75.

Using the estimated VEs from Equations (2) and (3), we further investigated the relationships between VEs and HDI across countries. We found that the HDI has a weak correlation with short-term VEs (Spearman correlation coefficient *r*_s_ = −0.33, *p* < 0.01) and long-term VEs (*r*_s_ = −0.21, *p* < 0.01). Additionally, we discovered that countries with the highest HDI have greater effectiveness in both short-term and long-term VEs, followed by the effectiveness of the group of HDI Q3 (Figure 5).

Then we found that HDI was positively related to the rate of people vaccinated (*r* = 0.80, *p* < 0.01) and people fully vaccinated (*r* = 0.83, *p* < 0.01) among countries (Supplementary Figure 3). These results imply that countries with a high HDI are likely to have a high VCR, which leads to a more effective vaccination impact. While the relationships were weak, it is possible that some countries with low or moderate HDI also presented suitable vaccination effects.

### Distribution of Position Between the Incidence Rate, Average GSI, and VCR Among Countries

**Figure 6:**
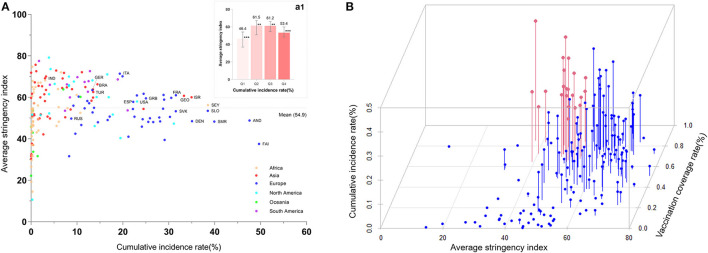
Distribution of the position between cumulative incidence rate, vaccination coverage rate, and average stringency index among countries. In part **(A)** the number denotes median and error bars denote quantiles 0.025 and 0.975. *** means the group has significant differences from the other three groups, and ** means the group has a significant difference from the other two groups. **(A)** shows that countries with high and low incidence rates both had a lower GSI, whereas countries with a medium incidence rate (Q2 and Q3 groups) had the highest GSI. Dots with red color in the **(B)** represent the countries with higher VCR but still a high incidence due to a lack of appropriate GSI.

We examined the distribution between cumulative incidence rate and the average GSI among countries through a two-dimensional graph. Figure 6A shows that countries with a low incidence rate (<0.63%) has a lower GSI, while countries with higher cumulative incidence rates (>14.47%) retain a lower GSI. The highest GSIs were found in countries with incidence rates ranging from Q2 to Q3 (0.63–14.47%). Six of 10 countries with the highest incidence rates (Faeroe Islands, Andorra, San Marino, Slovenia, Denmark, and Slovakia) had GSIs lower than the global average of 54.9.

Furthermore, we plotted all the countries on a three-dimensional graph (Figure 6B) using the average stringency index, cumulative incidence, and VCR as three axes and observed the corresponding position of each country. We found that there are some countries (highlighted by the red color) that have a greater VCR (>60%) but a higher incidence (>14.47%) due to the comparatively low GSI (<54.9).

## Discussion

To the best of our knowledge, this is the first study to examine the impact of vaccination and government stringency control measures on the COVID-19 incidence using long-time global data (108 weeks) worldwide. We illustrated that vaccinations were generally effective in reducing disease incidence worldwide. The effectiveness was better in Europe, North America, and Africa than in Asia, South America, and Oceania. Countries with a high HDI usually have a high vaccination coverage, resulting in a better vaccination effect. Nonetheless, some countries with high vaccination coverage did not receive a relatively low incidence. We argued that the primary reason for the inconsistency was a lack of appropriate GSI.

Since the first COVID-19 case emerged 2 years ago, SARS-CoV-2 has given rise to 12 different mutant strains from Alpha to Omicron (3), with Omicron causing a massive increase in incidence. Despite all these variants, vaccination continued to provide a reasonable level of protection against SARS-CoV-2 infections worldwide. The effect of vaccination was gradually increased and peaked roughly 2 months later, followed by a slow drop. These findings are consistent with those of laboratories and local populations (2, 4, 5, 7, 10, 30).

Europe, North America, and Africa had a higher VE than other continents. There could be two explanations for this, which are as follows: (1) Europe and North America had higher VCR of 64.0% and 61.0%, respectively, both of which were significantly higher than the global average (54.2%). Although VCR in Africa was low (11.5%), its incidence rate was similarly low (0.8%), resulting in a comparably better VE. (2) The fact that the efficiency of vaccine manufacturers differs may also be a factor. The primary types of vaccines currently accessible in Europe and North America were manufactured by Pfizer/BioNTech, Moderna, and Oxford/AstraZeneca, which were reportedly more effective than other similar vaccines in the global market (31).

Countries with a high HDI have a higher VCR (*r* = 0.80, *p* < 0.01), but this does not guarantee a low incidence rate. This could be attributed to three main reasons: (i) countries with high HDI have desirable economic security conditions and hence are usually able to maintain high VCR, but this does not imply that they have effective government stringent measures. (ii) Countries with a medium HDI (0.59–0.85) had a higher GSI (*p* < 0.05), whereas countries with a high HDI (>0.85) had a lower GSI. Additionally, GSIs of countries with high COVID-19 incidence rates (>14.47%) were lower than those with a medium incidence (0.63–14.47%). (iii) HDI was positively associated with COVID-19 infections (*r* = 0.81, *p* < 0.01; Supplementary Figure 4), indicating that the higher a country's HDI, the greater the likelihood of disease infection, and vice versa. This further suggested that infections are not always better controlled in countries with a high HDI, which is consistent with recent research (32) on the “hygiene hypothesis.” Countries with low socioeconomic status and poor sanitary conditions reinforced the population's innate immune system as a result of frequent exposure to microorganisms in the environment, which may somehow reduce the population's sensitivity to SARS-CoV-2.

Notably, the top 10 countries with the largest populations had comparatively lower incidence rates (Figure 6A), possibly because the bigger population is calculated as the denominator for the incidence rate. More importantly, most of them have implemented stricter control measures primarily to avoid more infections (GSIs of 9 countries are higher than the world average), which would subsequently have a greater burden on the economy than countries with a smaller population.

There, if countries have a high vaccination coverage that has reached a certain threshold (e.g., 69%, estimated from R_0_ = 2.5) to form the herd immunity among the population, the GSI should gradually be loosened to revive the economy. Suppose countries still have a low VCR that is far below the immunity herd; in that case, it is preferable to maintain a certain level of government stringent measures until the vaccination coverage achieves immunity herd levels.

Some limitations must be acknowledged. Firstly, this paper is a global data-based analysis, and there will be some country-specific data variations. Even though several methods and techniques have been adopted in the data source sectors to minimize differences and ensure data consistency, there may still be some variations between countries, such as differences in the methods for detecting confirmed cases, discrepancies in vaccine manufacturers and vaccine dispensing, and heterogeneities in extensive cultural, political, and environmental factors. Secondly, re-infections in the proportion of new cases, although sporadic and occur at a low rate, may affect the vaccine's effectiveness in the analysis to some extent. Thirdly, GSI data are generally collected from the sources of major media releases and the internet, which are rough approximations and do not fully reflect the actual situation of governmental response to disease surveillance and control. Moreover, there is a lag between the GSI and the change in disease cases. Although we set the lag = 1 week for GSI according to the literature during the analysis, the lag value varies across countries. Lastly, our study was performed on a global scale, and thus the estimators should be interpreted cautiously at the regional level.

In conclusion, Europe, North America, and Africa had a higher level of VE than Asia, South America, and Oceania. The long-term effects of vaccination outperformed the short-term effects. Countries with a high HDI usually have a high vaccination coverage, leading to a better vaccination effect. However, some countries with high vaccination coverage did not have a relatively low incidence due to a relatively weaker GSI. Thus, in addition to increasing population vaccination coverage, it is crucial to maintain a certain level of government stringent measures in preventing and controlling COVID-19 infections. The strategy is particularly suitable for countries that currently have low vaccination coverage.

## Data Availability Statement

The raw data supporting the conclusions of this article will be made available by the authors, without undue reservation.

## Ethics Statement

Ethical review and approval was not required for the study on human participants in accordance with the local legislation and institutional requirements. Written informed consent from the participants' legal guardian/next of kin was not required to participate in this study in accordance with the national legislation and the institutional requirements.

## Author Contributions

LS and KL conceived, designed, and supervised the study. PY prepared the first draft of the manuscript. ZY analyzed the data and prepared the figures. ZS, XL, and CZ provided critical revisions of the manuscript. All authors read and approved the final manuscript.

## Funding

This work was supported by the National Natural Science Foundation of China (82173627 and 81803289), the Logistics Science and Technology Youth Cultivation Program (20QNPY047), and the Medicine Enhancement Programme of FMMU (2020SWAQ10).

## Conflict of Interest

The authors declare that the research was conducted in the absence of any commercial or financial relationships that could be construed as a potential conflict of interest.

## Publisher's Note

All claims expressed in this article are solely those of the authors and do not necessarily represent those of their affiliated organizations, or those of the publisher, the editors and the reviewers. Any product that may be evaluated in this article, or claim that may be made by its manufacturer, is not guaranteed or endorsed by the publisher.

## Supplementary Material

Supplementary Figure 1 Short-term and long-term effects of vaccination on cases between two types of variants among continents. Parts **(a)** and **(b)** show the effects on cases of Alpha, Beta, Delta, etc variants. Parts **(c)** and **(d)** show the effects on cases of the Omicron variant. Red circles are filled if the value is significantly different from the null distribution (*p* < 0.05), and open otherwise. The number denotes the median, and error bars denote quantiles 0.025 and 0.975.

Supplementary Figure 2 Number of countries of short-term **(a)** and long-term **(b)** effects. Part **(a)** shows that proportions of countries being effective were higher in Europe and North America than that in Africa and Asia. Part **(b)** reveals that the proportions were higher in Europe as well. All countries showed significantly effective on vaccination by Equations (2) or (3).

Supplementary Figure 3 Human development index had strong positive correlations with the people vaccinated rate **(a)** and people fully vaccinated rate **(b)**.

Supplementary Figure 4 Relationship of human development index (HDI) and cumulative incidence rate among countries. The figure reveals that HDI had a positive correlation with cumulative incidence rate among countries. The number denotes the median, and error bars denote quantiles 0.025 and 0.975. ^***^ means that the group had a significant difference from the other three groups.
