# Supplementary figures and images for: Vaccination and Government Stringent Control as Effective Strategies in Preventing SARS-CoV-2 Infections: A Global Perspective
Source: Front Public Health. 2022 Jun 24;10:903511. doi: 10.3389/fpubh.2022.903511 (PMC9263831; doi:10.3389/fpubh.2022.903511)

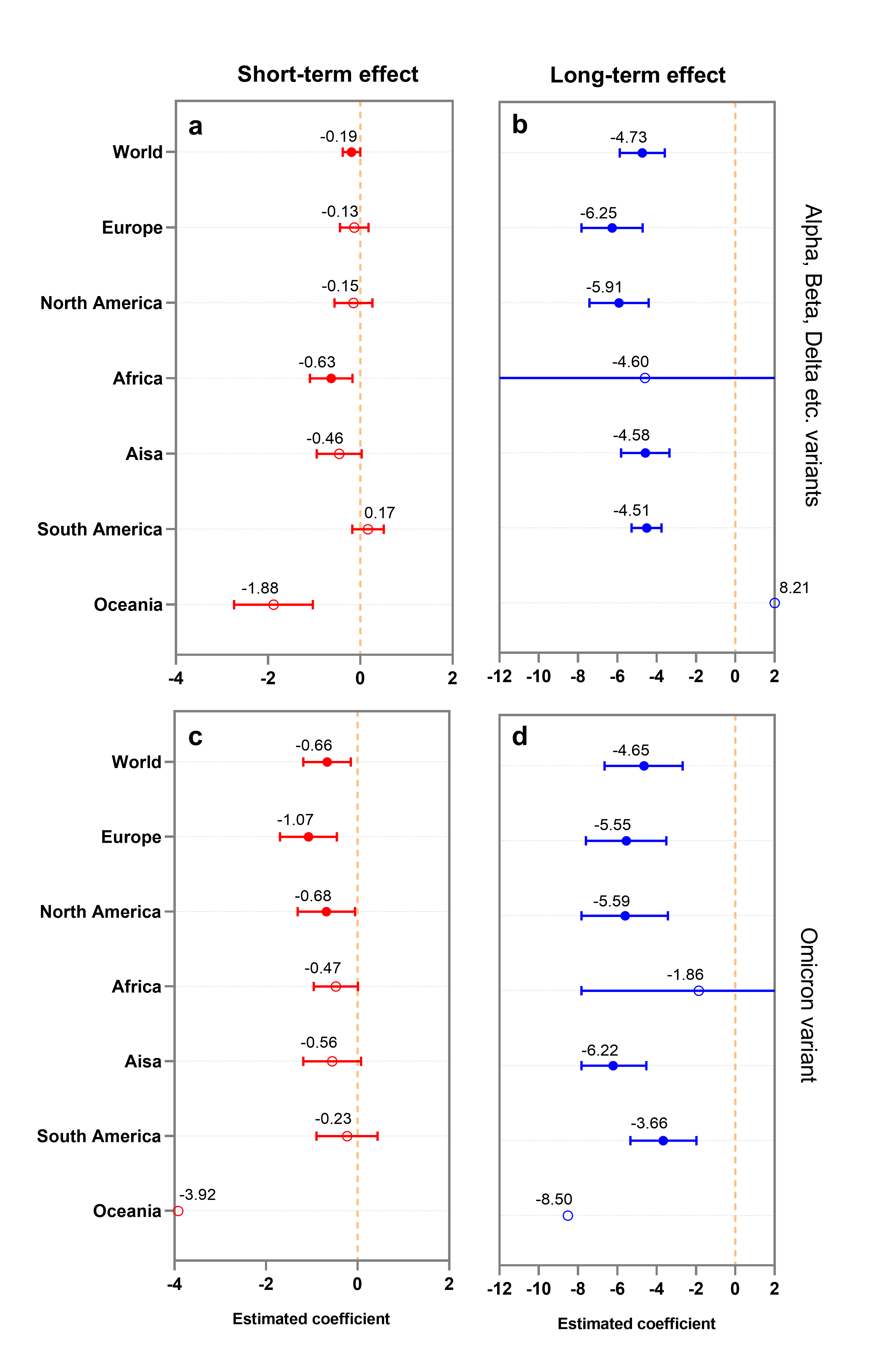

Supplement: Supplementary Figure 1 — Short-term and long-term effects of vaccination on cases between two types of variants among continents. Parts (a) and (b) show the effects on cases of Alpha, Beta, Delta, etc variants. Parts (c) and (d) show the effects on cases of the Omicron variant. Red circles are filled if the value is significantly different from the null distribution (p < 0.05), and open otherwise. The number denotes the median, and error bars denote quantiles 0.025 and 0.975. [file Image_1.TIF]

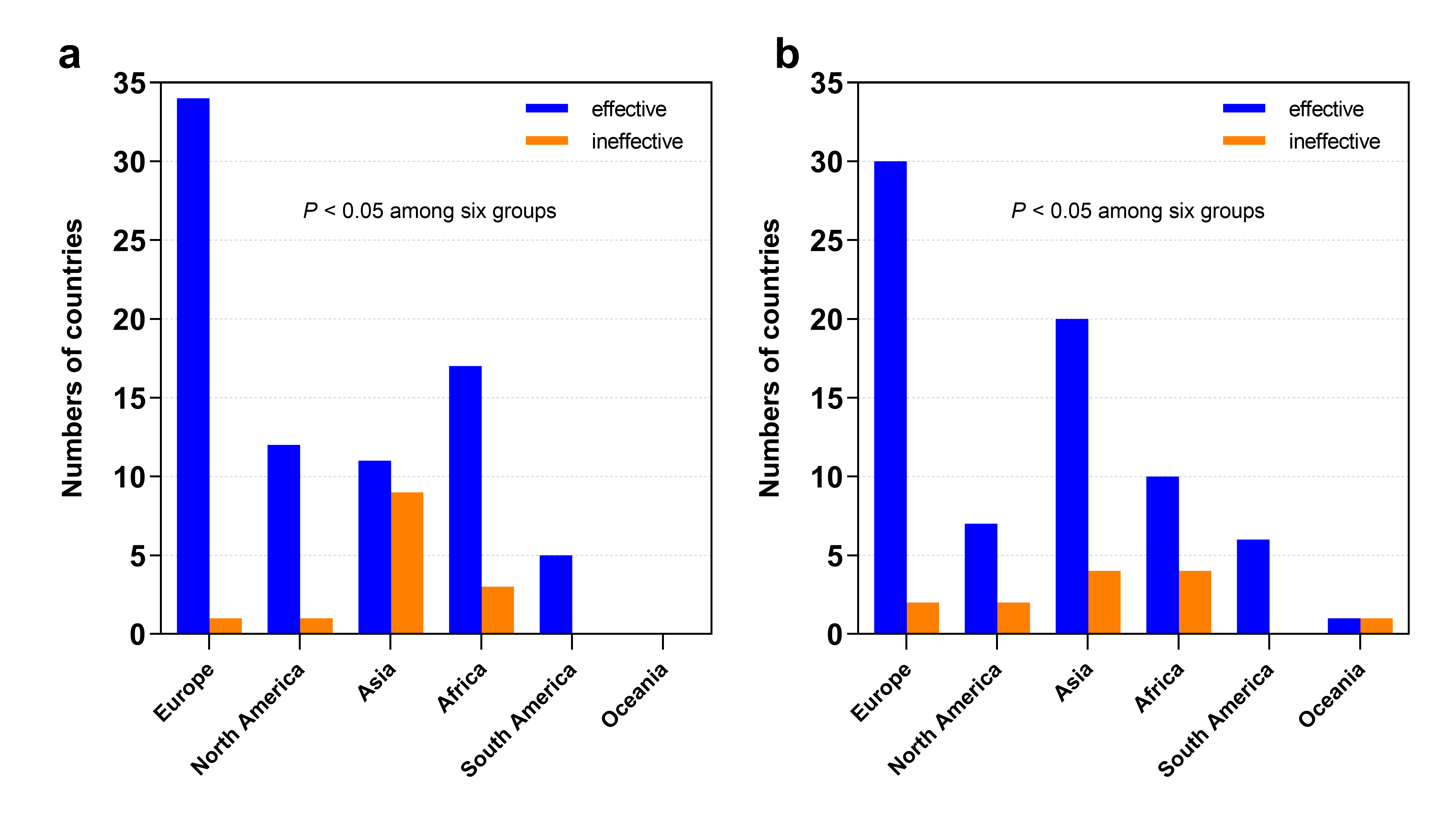

Supplement: Supplementary Figure 2 — Number of countries of short-term (a) and long-term (b) effects. Part (a) shows that proportions of countries being effective were higher in Europe and North America than that in Africa and Asia. Part (b) reveals that the proportions were higher in Europe as well. All countries showed significantly effective on vaccination by Equations (2) or (3). [file Image_2.TIF]

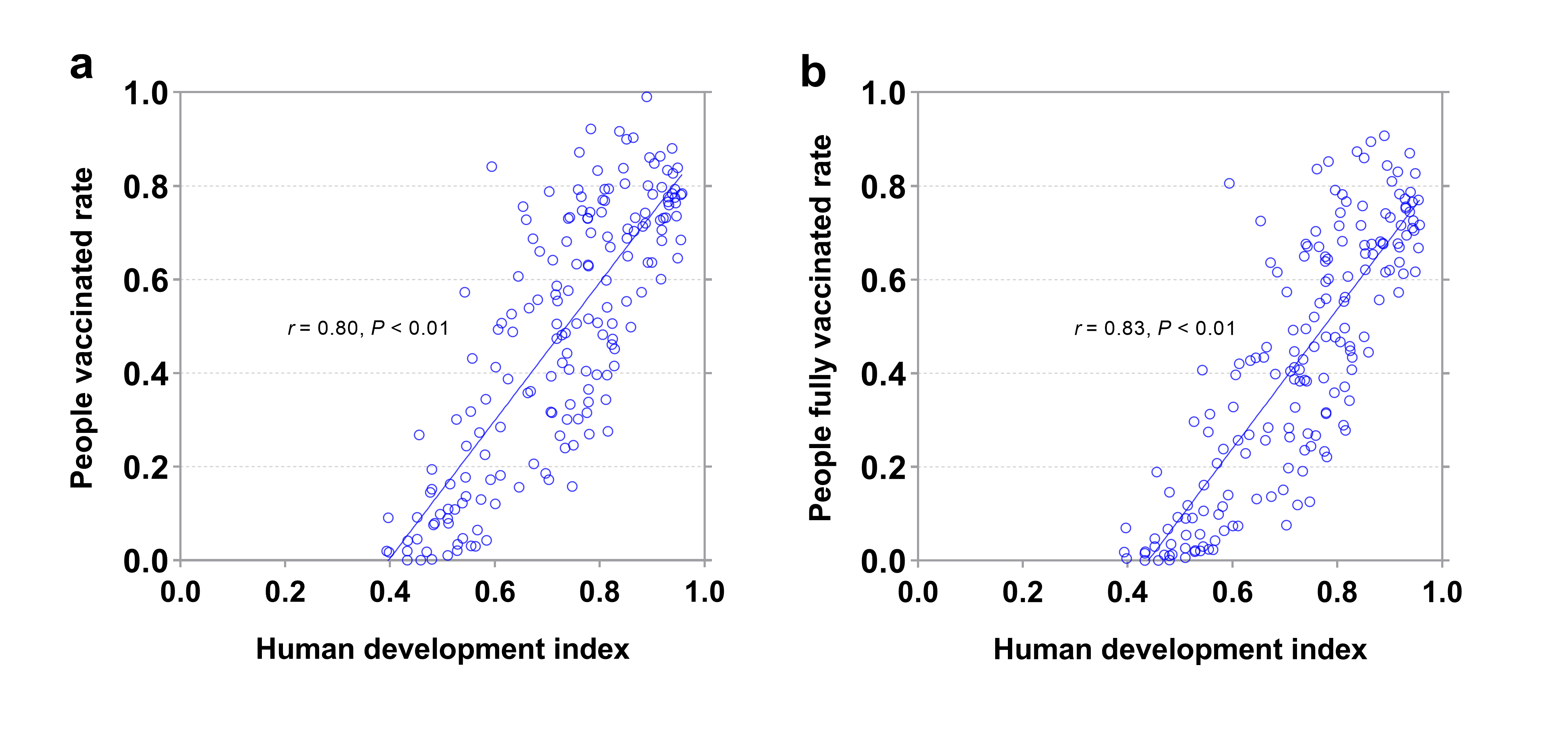

Supplement: Supplementary Figure 3 — Human development index had strong positive correlations with the people vaccinated rate (a) and people fully vaccinated rate (b). [file Image_3.TIF]

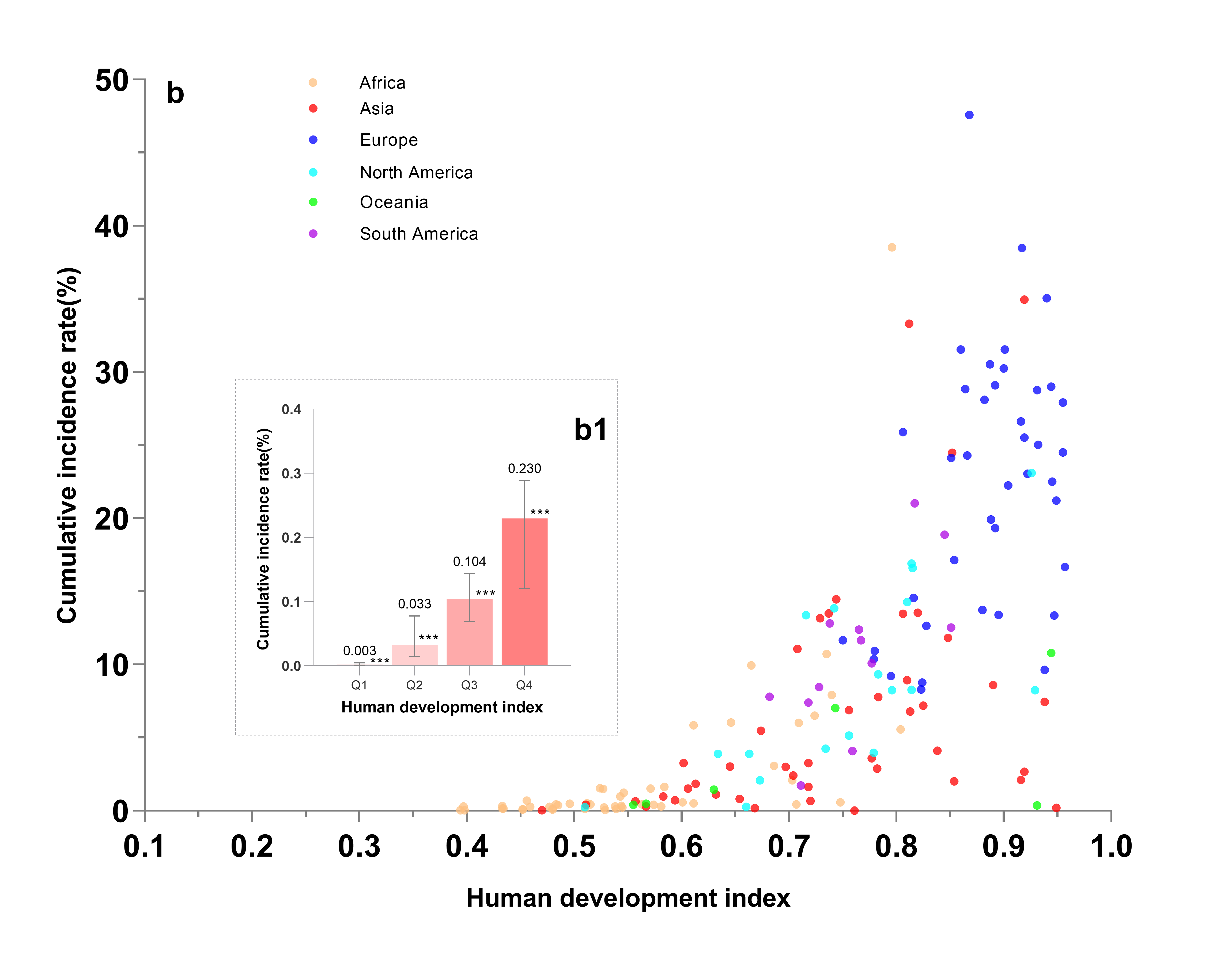

Supplement: Supplementary Figure 4 — Relationship of human development index (HDI) and cumulative incidence rate among countries. The figure reveals that HDI had a positive correlation with cumulative incidence rate among countries. The number denotes the median, and error bars denote quantiles 0.025 and 0.975. *** means that the group had a significant difference from the other three groups. [file Image_4.TIF]
